# Nurse management of minor problems in primary care emergencies: a non-randomized controlled trial

**DOI:** 10.1186/s12912-025-02729-2

**Published:** 2025-01-24

**Authors:** Jordi Estarlich-Corominas, Neus Soler-Abril, Anna Casanellas-Chuecos, Sara Becerra-Corzo, Andrea Sofia Bianco, Pere Torán-Monserrat, Rosa García-Sierra

**Affiliations:** 1 Institut Català de la Salut, Barcelona, Spain; 2 Unitat de Suport a la Recerca Metropolitana Nord, Fundació Institut Universitari per a la recerca a l’Atenció Primària de Salut Jordi Gol i Gurina (IDIAPJGol), Mataró, Spain; 3 Department of Medicine, Faculty of Medicine, Universitat de Girona, Girona, 17004 Spain; 4 Faculty of Medicine, Department of Nursing, Universitat Autònoma de Barcelona, Barcelona, Spain

**Keywords:** Health services needs and demand, Emergency medical services, Delivery of health care, Primary nurse, Minor problems, Primary health care, Nursing care

## Abstract

**Background:**

Health systems must implement strategies to adapt to the high demand in primary care caused by social changes. Since 2009, the Catalan Institute of Health has been expanding the Nurse Demand Management programme through which nursing autonomously addresses minor health problems. This study aims to analyse whether this programme is a factor in improving the efficiency and quality of care in a primary care emergency centre. The results come from a non-randomised controlled experimental study that tested the implementation of the programme applied to an experimental group treated by nurses versus a control group treated by doctors. The study was approved on 14 July 2022 by the Research Ethics Committee of the Jordi Gol University Institute following the guidelines of the TREND statement.

**Results:**

A total of 312 patients were included in the study, all of whom came to the emergency centre with five types of minor problems. Exactly half (156) were seen by nurses and half were seen by doctors. The experimental group had an average waiting time of 15.1 min and the control group 33.25 min. There was no significant difference in the assessment of the quality of care received and there were no differences in the total number of consultations for the same problem. There were fewer prescriptions given out by nurses, with an average of 1.79 medicines per participant versus 2.26 medicines prescribed by doctors. The results of the study suggest that the Nurse Demand Management programme was a factor in improving the efficiency and in the emergency centre. Nurse resolution capacity, after applying algorithms, offered quality care, with fewer prescriptions and good patient satisfaction without increasing the total number of consultations due to the same problem.

**Trial registration:**

The protocol for the current study was registered at Clinicaltrials.gov, with identification NCT06298240, retrospectively registered February 29, 2024.

**Supplementary Information:**

The online version contains supplementary material available at 10.1186/s12912-025-02729-2.

## Introduction

Demographic and technological changes, an aging population, and changing health expectations, are common social elements in many countries that determine how citizens experience everything related to health and the processes of getting ill. This in turn affects how primary care (PC) services are used. Other characteristics of society today, such as a prevailing consumerist culture and the medicalization of health, which influence the frequency of consultations [1, 2] must not be forgotten. These challenges, coupled with the increase in chronic diseases and multimorbidity, along with the prevention through the promotion of healthy lifestyles and the substitution of hospital care with community-based care, have led countries around the world to develop new models of primary care delivery [3].

According to the primary health care activity monitoring programme Primary Health Care Activity Monitor for Europe (PHAMEU), funded by the European Commission and operating in 31 countries, PC is currently facing challenges, regardless of the delivery model, derived from a change in demand by the population, a change in the healthcare offer and changes in the financial and technological context [4, 5].

Furthermore, the global economic situation forces governments to reformulate the financing of health services that are under pressure, mainly due to socio-demographic changes. Among the reasons for the increased pressure on health services are the high rates of visits to PC. This is a phenomenon that has been covered by diverse literature from the last two decades [6, 7] and which has been conceptualized as a small subgroup of patients who consume most primary care services [8]. Care of emergencies coming to PC is a challenge in all health systems. In the United States, they amounted to 22% of the 1,090 million outpatient visits per year between 2001 and 2004 [9]. This increasing rate of consultations involves a high cost for public health systems, and a considerable workload and much frustration for general practitioners [10]. In this cross-sectional study conducted in Catalonia, Spain, based on 3,815 interviews with patients from 77 primary care centres, researchers linked frequent visit attendance to factors such as obtaining sick leave, reporting mental health problems, arthritis/rheumatism or bronchitis, among others. Furthermore, and according to a review of 61 articles, mostly from the United Kingdom, the United States and Scandinavia [11], 10% of the patients who attend most frequently represented between 30% and 50% of all consultations.

Health care pressure in primary care has required the reorganization of health professionals and their roles. In this global challenge, the historical evolution of knowledge in the nursing profession based on models and theories of care that support its practice, has abandoned the dependence that characterized it initially as a discipline of support for tasks complementary to medical work [12], in order to build the science of nursing [13]. This science, with a variety of definitions according to each of the theories that the history of nursing has been building, is the substantive knowledge specific to the discipline that focuses on the human health process articulated in nursing and which, according to each theory and its definitions, have as aims helping people to achieve, maintain and recover health (Rey Imogene), facilitate optimal well-being (Betty Neuman), compensate or overcome known or emerging health problems (Dorotea Orem) or promote human improvement in whatever state the person is found to be (Martha Rogers) [14]. It is from this science that nurses currently apply scientific reasoning to their nursing process for patient-centred care, by applying the five sequential steps: assessment, diagnosis, planning, implementation and evaluation [15]. The role of nursing and improvements in training are becoming valid tools for addressing the field of minor health problems. However, international differences in the training of nurses make it difficult to standardize care models due to a disparity in their competencies. In the United States, their advanced training and autonomous roles mean the provision of quality chronic care for patients with pathologies such as diabetes, hypertension, arthritis or asthma can be given, thereby achieving a reduction in the rate of hospital admissions [16]. It must be underlined that there is no data to suggest that nursing professionals in states that impose greater restrictions on their competencies provide safer and better care than those in less restrictive states [17]. These authors state that eighteen US states have liberalized and standardized legislation to expand independent practice and prescribing by nurses. Furthermore, and added to the capacity for nurses to address patients’ health problems, it is necessary to take into account the international evolution of prescribing medicines. Today, nurses can legally prescribe medications in quite a few countries, in some cases under specific circumstances; examples include Australia, Canada, Finland, Ireland, New Zealand, Norway, South Africa, Sweden, the Netherlands, the United Kingdom and the United States of America [18].

Over the last decades, several studies have compared the health care of doctors with that of nurses. A meta-analysis looked at care provided by nurses or midwives in primary care settings in the United States and Canada, with that provided by doctors. The results showed that the care provided by nurses achieved better treatment adherence and satisfaction [19]. Along the same lines, a more recent systematic review [20] indicated that nurses’ care was comparable to that of doctors, but also that nurses obtained better levels of satisfaction and spent more time on seeing patients.

The latest published systematic review by Laurant et al. [3] identified 18 randomized trials that showed that for some persistent physical ailments and chronic diseases, trained nurses provide equal or even better quality care than that offered by primary care practitioners, and they may even achieve better health outcomes.

In Spain, a four-year university degree is required to practise as a nurse, sufficient training to be able to autonomously and safely care for patients with minor acute illnesses [21]. Regional and national legal frameworks have also developed significantly since 2015 with regard to the ability of Spanish nurses to prescribe medications. Royal Decree 1302/2018 reoriented the state legal framework for the indication, use and authorization of dispensing by nurses with respect to certain medications subject to medical prescription through a marked collaborative nature and with the aim of trying to guarantee continuity of care and safety of any patient, and, on the other hand, modifying and expanding the requirements demanded to nursing in order to be able to prescribe.

In Spain, the bulk of health-related competences are transferred to its 17 autonomous communities, a progressive process carried out over 35 years that ended in 2001 [22]. Catalonia was one of the first regions to manage its health services. The Spanish government transferred these competences in 1981 through Royal Decree 1949/1980.

In the early 1990s, the Catalan Institute of Health (ICS), the largest public health services entity in Catalonia, began a process to maximise the professionalization of primary care nurses with the aim of adapting their services to the needs of the population. From that initiative emerged the first manual of standards for nursing diagnoses in PC [23]. This first initiative was updated in 2003 with the aim of facilitating the application of the nursing care process as a systematic method for planning and providing nursing care. Between 2005 and 2006, the ICS implemented the standardization of nursing diagnoses with the NANDA classifications, NIC nursing interventions and NOC (NNN) outcomes [24, 25]. Finally, in 2018, as a result of a broad internal study of the perception and opinion of PC nurses, it was evident that NNN language ​​has an insufficient level of clarity, ease of use and utility. For this reason, care plans are updated based on ATIC terminology [26], a Spanish acronym for Architecture, Terminology, Interface-Nurse-Information and Knowledge, currently in use in PC in Catalonia [27].

In parallel with this period of change, in 2009 the Catalan Department of Health promoted several measures aimed at improving the health system. The Primary Care and Community Health Innovation Plan involved a reorganization of the PC [28]. The objective was to improve economic sustainability and meet the growing demand of patients by referring them to the professional who could best respond within their area of ​​specialization [29]. At this stage, the gradual opening of Primary Care Emergency Centres (PCEC) began in strategically demographic points within Catalonia, intended to address minor and non-serious health problems, open 24 h a day, 365 days a year, and supporting both primary care and hospital emergency services. Within this period of healthcare reorganization in Catalonia, the concept of Nurse Demand Management (*NDM*) was born [30], a programme led by primary care nurses to address minor health problems. A set of health problems such as minor emergency situations that may be potentially complex, but without life-threatening risk, or non-urgent situations that allow for delayed care and can be scheduled without risk to the patient [31]. This programme is based on agreed protocols and the standardization of diagnoses where the nurse can resolve this demand without the intervention of a doctor and within their area of ​​expertise. In 2016, the ARES-AP (Treatment Standards Harmonization Program) computer tool was deployed in PC [27, 32], an application that collects all the standardized treatment plans. It is designed using ATIC language for nurses to use within their nursing process methodology, in which they make clinical decisions aimed at identifying and resolving or preventing health problems, based on the assessment, diagnosis, planning, execution and evaluation of health problems [25].

In Catalonia, and to the best our knowledge, there has been only one randomized trial comparing the effectiveness of care provided by nurses with the usual care provided by general practitioners. In that study, the nurses demonstrated a capacity for resolution, with success in 86% of the cases while no differences were observed in the resolution of symptoms or satisfaction of patients between groups [33]. This study was carried out before the implementation of the NDM.

Thanks to the results of the implementation of the NDM and the public satisfaction rates, currently 30 paediatric and 36 adult protocols are active in the computerized clinical history software programme (ECAP) of the Catalan Institute of Health.

In the Catalan area, the literature offers results from various transversal studies that agree on the ability of Catalan nurses to solve minor health problems, among which we highlight, on the one hand extensive multi-centre retrospective work by Fernández Molero et al. [34] that included data from medical records between 2019 and 2020 and in which 392 primary care areas of the Catalan Institute of Health participated. In this case, the NDM protocols were fully in force and minor health problems resolved by nurses were 50.9% of adult patients and 55.4% of paediatric patients. However, one of the limitations of this study, which the authors recognized, was not recording the rate of other consultations afterwards for the same reasons. This is a variable that we consider a key element in evaluating the resolution capacity of the NDM. On the other hand, we also highlight the longitudinal study with a 3-year follow-up carried out by Jurado-Campos et al. [35] in which they showed resolved cases increased and the number of other consultations afterwards decreased, concluding that nursing capacity for managing the demand of patients without an appointment improved following an interdisciplinary intervention using a mutually agreed upon and locally adapted approach.

The objective of this study was to determine if the implementation of the NDM in a PCEC improved the efficiency and quality of care.

## Method

The Primary Care Emergency Centre (PCEC) serves a population of 270,000 inhabitants of the Maresme region, in the province of Barcelona, in the autonomous community of Catalonia, Spain [36]. The PCEC is located in the municipality of Mataró and started operating in May 2021. Since it opened, it has seen more than 100,000 patients, with it being considered a key piece within the territory’s health system. Its management depends on the Catalan Institute of Health. Potential participants for this study were patients assigned to the demographic area of influence of the health centre.

The protocol for the study was registered retrospectively registered February 29, 2024 in the CinicalTrials.gov (CT) database of the US National Library of Medicine (NIH) with registration number NCT06298240.

### Design and participants

A non-randomised controlled experimental study was developed, following the TREND statement guidelines [37], carried out in a PCEC, where the NDM procedure was applied. This procedure was carried out by volunteering nurses who cared for patients at the centre during their working hours, applying the NDM. The participation of nurses and doctors in the study was not financially compensated and involved 6 volunteer nurses out of the 14 who were previously trained in the NDM procedure. They were assisted by the 3 doctors on duty at the emergency center. These professionals combined their participation with their daily tasks. The nurses were organized into shifts to perform the NDM, but they also managed patient triage based on priority and severity, administered treatments and medications, and conducted blood tests, among other duties. The doctors saw patients with varying levels of priority and complexity, from the highest to the lowest. The nurses were responsible for informing patients about the study and providing the informed consent documents and post-visit satisfaction surveys to those who agreed to participate, both in the control group (assessed by doctors) and the intervention group (assessed by nurses).

During September 2022, the volunteer nurses received printed theoretical material with the five algorithms accepted for this study, non-complex respiratory tract problems, mild urinary problems, toothache, minor wounds and gastrointestinal disorders. They also had digital access to the algorithms or care plans, through the ARES-AP nursing care computer program. In the same period, these nurses also participated in one of the two practical clinical assessment sessions given by a volunteer doctor from PCEC.

1. The inclusion criteria for the study were:
  Presenting any of the following health problems:
  Non-complex airway issues (rhinopharyngitis or common cold, pharyngitis, tonsillitis or respiratory virus).
  Urinary problems (cystitis or urinary tract infection, only in women).
  Acute skin lesions (burns or acute wounds).
  Toothache.
  Gastrointestinal disorders.
2. Being between 16 and 65 years old.
3. Visit the PCEC between October 2022 and February 2024.
4. Having signed the written informed consent.

An exclusion criterion was presenting a screening with a priority of care lower than 3, according to the Andorran screening model (*MAT*), a preliminary clinical assessment process that sorts patients before diagnostic and therapeutic evaluation based on their degree of urgency until they can be seen by medical or nursing disciplines. This is a structured triage computer system created for emergency services in hospitals or PC centres, developed from the year 2000 in Andorra, as an evolution of the Australian-Canadian school of triage [38, 39] and which is integrated into the Catalan emergency plan [31]. Priority care screening is the first care received by all patients who come to PCEC, regardless of whether they are subsequently treated by doctors or nurses. Nurses apply and assign the patient a priority level for urgent care. Grades less than 3 are assigned to foreseeable life-threatening situations (level 2), or situations requiring immediate life-threatening resuscitation (level 1). Levels ≥ 4, which correspond to the less serious cases of patients with minor health problems, can be treated autonomously by nursing through the treatment plans provided in the PC [32] or by doctors, according to the organizational needs of the PCEC.

An estimate of the sample size was made based on the variable waiting time for the visit. Considering a waiting time of less than 15 min for 50% of the participants, to detect as statistically significant a 20% increase in participants in the intervention group, a sample size of 156 participants in each group (*N* = 312) was estimated to be necessary. An alpha risk of 0.05 and a beta risk of 0.1 in a bilateral contrast were accepted for this estimation.

### Assignment

The assignment to the groups was made based on the fortnight of the month in which they attended the PCEC. Patients who were seen during the first 15 working days of the months of study between 10 a.m. and 8 p.m. were assigned to the experimental group, in which the NDM intervention was applied. The participants in the control group, treated with the usual medical procedures, were assigned during the second fortnight of the months of study at the same time and on the same workdays. The reason for carrying out this assignment was to avoid cross contamination or interference between the two groups.

### Intervention

The NDM intervention consisted of applying to an experimental group of patients a programme based on agreed protocols according to scientific evidence and which in this study was specified with five algorithms to treat non-complex respiratory tract problems, mild urinary problems, toothache, minor wounds and gastrointestinal disorders. These algorithms were applied autonomously by trained nurses to 156 patients who consulted for one of the five reasons and who agreed to be treated with this procedure.

### Variables

The variables needed for the study were taken on the same day as the visit and one month after it. This included the time of arrival at the PCEC, the time of the start of the consultation and the time of departure. Patient satisfaction data was also collected through an anonymous paper questionnaire that was completed after leaving the doctor or nurse.

One month after the visit, data was extracted from the computerized clinical history of the participants related to demographic variables, health history or prescription of chronic medicines, possible subsequent consultations for the same reason in the same centre or in other primary care centres or hospitals, the number of medicines prescribed to treat the reason for the consultation and the need for sick leave. A second questionnaire was also conducted by telephone to assess the patient’s overall satisfaction with the entire process.

The data was entered into the Research Electronic Data Capture REDCap^®^ management platform [40, 41], in a questionnaire created exclusively for this study. This data was recorded on the same day as the consultation and after the telephone satisfaction survey.

### Flows and recruitment

**Fig. 1:**
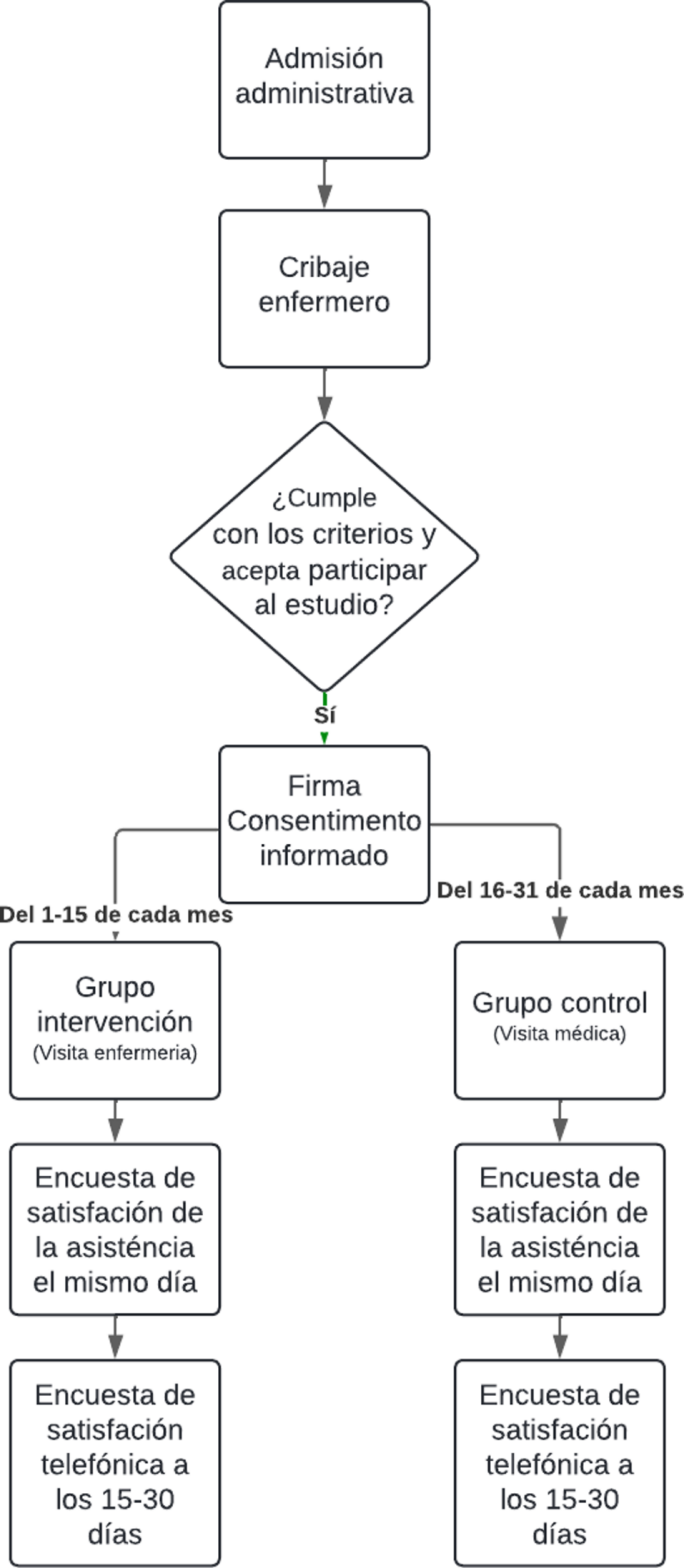
Diagram of flow and recruitment

Patients first interact with the PCEC in the triage consultation, where they were given a level of priority in care according to their severity. Once the PCEC patients were classified according to their severity and MAT care time, the nursing staff informed them of the study and the possibility of participating. The profile of a potential participant was a user with a MAT triage ≥ 3, men or women aged between 16 and 65 years, who did not exhibit a significant language barrier and whose reason for visiting was for non-complex respiratory tract problems, mild urinary problems in women (men were not included in the study because urinary pathology in men is considered more serious and requires medical attention), toothache, minor wounds or gastrointestinal disorders. A total of 312 patients signed the informed consent, of which 156 participants were assigned to the experimental group (intervention) and who attended the PCEC in the first 15 days of the month, and 156 were assigned to the control group (standard medical consultation) between day 16 and the last day of the month. At the end of the nursing or medical visit, the participant answered a brief quality survey and between 15 days and one month later, they received a second telephone satisfaction survey (see Fig. 1).

### Data analysis

Univariate analysis was performed with mean and standard deviation for quantitative variables and frequency and percentage for qualitative variables. The assumptions for each test were checked before conducting the tests. Normality was checked with Kolmogorov-Smirnov test. Equality of variances was checked with Levene’s test. Socio-demographic and clinical variables were compared with Student’s t-test and Chi square to evaluate the comparability of the study groups. The outcome variables waiting times and those related to patient satisfaction were compared with Student’s t-test. Finally, the percentage of waiting time less than 15 min in the two study groups was calculated. All contrasts were made with an alpha of 0.05. The SPP v.26 statistical package was used [42].

## Results

### Description of the sample and comparison of the groups

Between October 2022 and February 2024, a total of 312 voluntary patients were recruited, man and women between 16 and 65 years whose reason for visiting was for non-complex respiratory tract problems, mild urinary problems in women (only for women), toothache, acute wounds or gastrointestinal disorders. There were 172 (55.1%) women. The average age of the participants was 32.73 years (SD 12.48). In the experimental group, 0.6% were assigned a MAT screening priority value of 3, 61.3% a value of 4, and 38.1% a value of 5. In the control group, no participant was assigned a MAT level 3, 69.2% a value 4 and 30.8% a level 5. Priority percentages of both groups without statistical difference (*p* = 0.228).

The main reason for being seen was upper respiratory tract problems, with 215 cases treated (68.9%), followed by gastrointestinal disorders, 52 cases (16.7%), toothache with 26 cases (8.3%) and urinary problems in 19 cases (6.1%). No injury cases were seen. Table 1 shows in detail the comparison of the socio-demographic and clinical variables of the experimental and control groups. The results indicate that there are no significant differences between them (*p* < 0.05), except in the case of hypertension, which was more represented in the control group. (Table 1).

**Table 1:** Comparison of socio-demographic and clinical variables between the two study groups

	M (SD)		t*	*P*
	Experimental (*n* = 155)	Control (*n* = 157)		
Age	31.32 (11.5)	34.06 (13.1)	-1.913	0.057
Number of prescribed medicines	3 (2.73)	2.62 (2.47)	0.643	0.522
	**n (/%)**		**X** ^**2**^	***P***
MAT Screening			2.960	0.228
3	1 (0.6)	0		
4	95 (61.3)	108 (69.2)		
5	59 (38.1)	48 (30.8)		
Gender			1.950	0.172
Female	91 (59.5)	81 (51.6)		
Male	62 (40.5)	76 (48.4)		
Reason for consultation				
Upper airways	94 (60.6)	110 (70.1)	3.057	0.096
Gastrointestinal disorders	25 (16.1)	24 (15.3)	0.042	0.877
Toothache	14 (9 − 0)	12 (7.6)	0.197	0.687
Urinary problems	12 (7.7)	6 (3.8)	2.205	0.153
Medical history				
Diabetes	2 (1.3)	4 (2.5)	0.654	0.684
Hypertension	3 (1.9)	13 (8.3)	6.454	0.018
Prostate pathology	1 (0.6)	0	1.016	0.497
COPD	0	4 (2.5)	4.00	0.063
Asthma	4 (2.6)	9 (5.7)	1.940	0.257
Heart failure	0	1 (0.6)	0.990	1.00
Kidney failure	0	1 (0.6)	0.990	1.000
Obesity	12 (7.7)	8 (5.1)	0.911	0.365
Neoplasia	1 (0.6)	4 (2.5)	1.791	0.371
Depression	2 (1.3)	5 (3.2)	1.276	0.448

M = mean SD = standard deviation n = number t*= Independent t − test

### Impact of the intervention on waiting times

As explained in the Method section, two different time fractions were collected. The first, the time elapsed from the patient going to the centre and being seen, and the second, the time elapsed from the patient going to the centre and being discharged. In the experimental group the time elapsed until being seen was 15.1 min (SD 18.42) and in the control group it was 33.25 min (SD 34.11). Nurses completed care in an average of 16.46 min and doctors in 13.69 min. The total time spent in the centre, that is, from the time the patient arrives until the patient is discharged, was 29.19 min (SD 22.05) in the experimental group and 46.73 min (SD 35.35) in the control group. From these results, in all reasons for consultation, the waiting time and the overall time until discharge were higher for the control group with significant differences in all cases, except the waiting time for being seen for gastrointestinal reasons. The comparison of the waiting times, for the consultation or the total time spent in the health centre according to the reasons for the visit, is described in Table 2.

The percentages of participants who waited less than 15 min to be seen were calculated. In the experimental group there were 107 people, which represents 69% of the group. In the control group there were 54, equivalent to 34.4% of the group. As such, there was an increase of 34.6% of patients who waited less than the 15 min to be seen in the experimental group. The average attendance time was 13.95 min (SD 14.42). In the experimental group the average was 14.47 min (SD 17.47) and in the control group, 13.42 min (SD 10.49). These time differences were not significant (*p* = 0.562).

**Table 2:** Comparison of waiting times according to reason for visit

	Time until seen					Total time in centre				
	Total (*N* = 312)	Experimental (*n* = 155)	Control (*n* = 157)	t	*p*	Total (*N* = 312)	Experimental (*n* = 155)	Control (*n* = 157)	t*	*p*
Upper airways	24.432 (28.25)	15.8 (18.38)	32.08 (32.96)	-4.53	< 0.001	37.61 (29.98)	29.23 (21.99)	45.04 (34.01)	-4.093	< 0.001
Gastrointestinal disorders	26.09 (32.25)	18.22 (23.92)	34.60 (38.03)	-1.842	0.073	40.38 (32.68)	31.30 (23.29)	50.20 (38.57)	-2.119	0.041
Toothache	24.77 (33.64)	7.71 (6.8)	44.66 (41.41)	-3.056	0.010	41.19 (36.69)	26.07 (18.68)	58.83 (44.90)	-2.496	0.020
Urinary problems	13.47 (13.99)	7.153 (5.8)	27.16 (27.18)	-2.780	0.001	31.79 (25.82)	27.92 (25.15)	40.17 (27.54)	-0.959	0.351

M = mean n = number SD = standard deviation t*= Independent t − test

### Patient experience

The patient experience was measured at two moments. The first, immediately after discharge, where the global assessment concerning the attention received was 9.56 (SD 0.979) on a scale from 0 to 10. In the experimental group it was 9.81 (SD 0.71) and in the control group it was 9.33 (SD 1.14), this difference being statistically significant (*p* < 0.001). The second, one month after the visit, the same global assessment measurement was carried out concerning the care received by means of a telephone survey. Using this measure, the score obtained by the sample was 8.75 (SD 1.55). The experimental group obtained a score of 8.8 (SD 1.6) and the control group 8.6 (SD 1.4), with this not being a statistically significant difference.

In this second measure, seven specific aspects of care were also evaluated, shown in detail in Table 3. Six of the seven aspects evaluated showed no differences in the scores received by the two groups. Differences were seen in the last question, which referred to whether the participant perceived that they would have been better served by another professional. In this case, the control group responded more affirmatively than the experimental group.

**Table 3:** Patient experience one month after visit (responses extracted from the telephone survey)

	Total (*n* = 179)	Experimental (*n* = 97)	Control (*n* = 82)		
	M (SD)			t*	*p*
Global evaluation after 1 month	8.75 (1.55)	8.8 (1.6)	8.6 (1.4)	0.472	0.637
Flexibility of the consultation	7.94 (2.05)	8.19 (1.97)	7.65 (2.12)	1.734	0.085
Professionalism	9.14 (1.2)	9.16 (1.25)	9.11 (1.19)	0.244	0.808
Effectiveness of treatment	8.57 (2.06)	8.52 (2.09)	8.62 (2.03)	0.966	0.754
Health advice	8.95 (1.622)	8.87 (1.63)	9.06 (1.65)	0.607	0.470

	**n(%)**			**Chi-square**	
Needed another consultation for the same reason	28 (15.6)	17 (17.5)	11 (13.4)	0.569	0.538
The treatment was effective	148 (82.9)	82 (84.5)	66 (80.48)	0.313	0.651
Considers that the consultation would have been more effective if it was carried out by another health professional	54 (30.17)	15 (15.4)	38 (46.34)	26.85	< 0.001

M = mean SD = standard deviation n = number t*= Independent t − test

### Consultations for the same reason, incapacity for work and prescribed medicines

In addition to the data from subsequent visits reported by participants in the telephone survey (Table 3) subsequent consultations for the same reason, in the same PCEC or in a PC centre or hospital emergency room in the healthcare area of influence, were extracted from the ECAP computerized platform. A total of 41 patients were seen again, 13% of the total sample. This was 21 patients (13.46%) from the control group and 20 (12.82%) from the experimental group, a non-significant difference. Only 7% of the participants who were seen again, 10 patients in the control group (6.41%) and 12 patients in the experimental group (7.6%) required a change in diagnosis, a new pharmacological prescription or additional tests, a non-significant difference (*p* = 0.316). A total of 89 incapacities for work were processed, assigned to 28.52% of the total sample, 46 (29.48%) in the control group and 43 (27.56%) in the experimental group, with no statistical significance (*p* = 0.401). In the experimental group, an average of 1.79 medicines were prescribed in 279 prescriptions. In the control group, an average of 2.26 medicines were prescribed in 353 prescriptions, being this difference statistically significant (*p* < 0.001).

## Discussion

The objective of this study was to determine if the implementation of the NDM in a PCEC improved the efficiency and quality of care. The criteria used for the evaluation were waiting times, health care time, patient satisfaction and the consultation rate afterwards for the same reason.

This study focused on the care of patients who were classified as low severity and priority for care after the MAT triage process, which was mandatory and prior to any visit, medical or nursing, at the PCEC. In cases where patients were classified with a triage ≥ 3, they could be treated under the usual medical discipline, or according to the needs of the emergency service, care was provided with the NDM procedure, where nurses apply nursing science within their methodology of the nursing process through standardized treatment plans [32], designed from language used in ATIC [26].

Consultations made by nurses and doctors were of similar severity. Various literature confirms the ability of nurses to care for patients minor health problems, demonstrating a high level of care, similar to that of general practitioners. These are the conclusions of the randomized studies by (Kinnersley et al., 2000; Shun et al.,2000) [43, 44], which are confirmed in the studies carried out in Catalonia by (Iglesias et al., 2013; Fabrellas et al., 2011) [33, 45].

With regards to the reasons for consultation selected for our study, it is worth mentioning that during the recruitment totalled a prevalence of 21.86%. Upper respiratory tract problems were the most prevalent (acute laryngitis, upper respiratory tract infections, pharyngeal pain or acute pharyngitis, cough, acute streptococcal tonsillitis, tonsillitis, rhinopharyngitis, viral infection and COVID), with a rate of 11.93%. In second place, urinary problems (acute cystitis without haematuria or urinary tract infection) with 3.21%; in third place, gastrointestinal disorders (vomiting, diarrhoea or gastroenteritis and infectious or non-infectious colitis) 2.30%; and finally, toothache 0.77%. This prevalence data shows the impact of minor health problems on acute demand in PC and leads to reflection on the importance of reinforcing health education as a measure to reduce demand [46]. Following this line of discussion, addressing hyper-frequency and care pressure in PC is the task of all professionals, and necessarily implies adapting tasks to different professional profiles. In this regard, care for frequent problems with low clinical risk, preventive activity and control of chronic processes can be taken care of by nursing [47].

Regarding the specific results of the four reasons for being seen, upper respiratory tract problems were in the majority for the entire sample with 68.9%. This result is related to the winter months and the flu and COVID peaks of 2022 and 2023 and which, like other years, activated the usual mechanisms contemplated in the Comprehensive Emergency Plan of Catalonia (PIUC) to address these periods of greater demand due to respiratory pathologies. Gastrointestinal disorders accounted for 16.7%, toothache for 8.3%, and urinary problems for 6.1%. As far as wounds are concerned and which were contemplated in the initial project of the study, they were finally not used as it is understood in our emergency centre that they are consultations that nursing cares autonomously (simple sutures or care of non-complex acute wounds) and which are generally only addressed by doctors when there is a complication such as a fracture, functional loss, neuromuscular or venous-arterial issues, cellulitis, etc. Given this, and in order not to interfere with the usual dynamics of the centre, we decided to not use them in this study.

In relation to the time dedicated to the visit, although we detected that nurses spent an average of 65 s more on the visit than their medical colleagues, the results indicate that there is no statistical difference between both professionals. It is true that some studies have found that the time invested by nurses on consultations is greater than that invested by doctors. Furthermore, this extra time is perceived by patients as an increase in the quality of care [3]. At the same time, the review by Karimi-Shahanjarini et al. [48] detected, in the majority of the 69 studies analysed, that patients thought that nursing professionals were more accessible than doctors. For their part, doctors and nurses considered that this substitution, when done with a collaborative attitude, increased the quality and continuity of patient care.

However, the fact that the nursing time invested in solving minor health problems is similar to that used in the usual interventions until now, by doctors, would imply that the care carried out by nurses would not mean an increase in resources due to the fact that it is carried out by different professionals. This leads us to reflect on the potential of implementing a fixed NDM consultation in the future, as a way of improving patient care by better distributing the medical and nursing care burdens.

The results of the study in relation to the efficiency of the NDM, based on the waiting time criterion, show that in the consultations made by nurses the delay in being seen decreased for all reasons for the consultations. Nursing showed a clear difference with their medical colleagues regarding the waiting time for patients before being seen. The experimental group reported shorter wait times for their patients, with 69% who waited less than 15 min to be seen, compared to 34.4% of the control group, treated by doctors. Therefore, an increase of 34.6% was found for the NDM consultation.

In the second evaluation criterion, related to the quality of care based on the frequency of consultations for the same health problem in the subsequent 15 days, we analysed whether the treatments proposed by nurses and doctors were effective. To do this, we reviewed the computerized clinical records of all participants. Some 13% of the total sample visited again for the same reason. In this analysis we see similar quality results in both groups. A proportion of 12.82% of NDM patients visited for the same reason, and of these, only 7.6% required a modification of treatment, prescription or request for new tests. In the control group, the percentage of consultations for the same reason was 13.46%; of these, only 6.41% required changes with respect to the initial visit. In line with these results, we find those of the study carried out by Fabrellas et al. [45] that also detected low rates of return of patients from a nursing management programme for same-day consultation, with 16 reasons for adults and 7 in paediatrics, with a rate of 3% in adult patients seen for back pain, nosebleeds, skin lesions and burns and 2% in paediatrics, seen for skin lesions and burns.

Some 53% of the visits made for the same reason did not imply a change in diagnosis or relevant medicines compared to the first visit in either group. This result invites us to reflect on the social change that citizens are experiencing in their way of facing the process of becoming sick and the use of health services. On the one hand, the consumerist and immediacy culture of today’s society reinforces the over-medicalization of mild acute pathologies or situations of personal or work discomfort, etc. On the other hand, the social-healthcare reality which is characterized by an increase in the prevalence of comorbidity and fragility, linked to aging, with more informed patients, who want to participate in the decisions that affect their health, in an increasingly digitalized society [49]. The care of patients in primary care consultations is a multi-causal phenomenon influenced by the social environment, health organizations and professionals [50]. Healthcare organizations and their professionals must face these new challenges with more cohesive teams and new professional profiles.

Understanding the patient’s experience during the health care they receive, known as the patient journey [51], allows for the optimization of care processes and the detection of points that negatively impact their experience, and understanding their needs, demands and real concerns. To obtain greater knowledge of this experience, the participants were surveyed on the day of the visit, and also a month later. In the survey on the day of the visit, participant satisfaction was higher in the group treated by nurses (9.81) than in the group treated by medicine (9.33). The most plausible explanation for this result is linked to a shorter waiting time between priority care detection phase and being seen, compared to the consultation with a doctor, where the wait was longer. On the other hand, the survey carried out 30 days after the visit shows that there are no differences in satisfaction between the two groups. Two possible explanations for this change, produced by the passage of time, we believe may be, on the one hand, that the patient may have a clearer perspective 30 days after the visit, once the entire global process of the health problem has been completed; but, also, memory of the visit decreases after several weeks. There is little evidence on the effects of memory over long periods, but it can be expected that memory decreases over time and that respondents remember less after several weeks or months, as suggested by the work of Rettig et al., [52]. To reduce possible recall biases, at the time of the survey carried out one month after the visit, the patient was informed of details such as the date and time of the visit, the signing of the consent, the first quality survey answered, the reason for the consultation, the medication prescribed and whether there was any subsequent visit for the same reason in the clinical record. In this way, an attempt was made to refresh the patient’s memory in order to obtain a higher quality response. In this second survey, seven specific aspects of care were evaluated, looking for the strengths and weaknesses of the patient experience. The ratings of 6 of the 7 points were not different in the two groups, as shown in Table 3. However, the last aspect explored shows an unexpected result. To the question about whether the patients considered that the visit would have been more effective if it had been carried out by another health professional, 15.4% of the group seen by nurses responded that they believed that a doctor would have cared for them better; versus 46.34% of the group seen by doctors who considered that a nurse would have treated them as well as a doctor. This makes us think that patients are increasingly accepting nursing’s ability to treat minor health problems with quality. As we have mentioned in the introduction of this study, in Spain, a four-year university degree is required to practice as a nurse. Beyond this education, post-university nursing specialties are active, such as obstetrics and gynecology, mental health, occupational health, geriatrics, pediatrics, and family and community health, which require a two-year residency. In addition to the development of these specialties, Spanish nursing is also advancing in its collaborative role with doctors and other healthcare professionals, training and applying advanced clinical practice in areas such as case management in both hospital and community settings, home care [53, 54], or the management of chronic and acute patients in primary care [55]. All of this has a positive impact on the quality of healthcare patients receive and their satisfaction with nursing care.

Consistent with the results of our study, a multi-centre randomized controlled trial is cited that evaluated the acceptability and safety of a nurse-led minor illness service in the United Kingdom [44] where patients agreed with our study in being more satisfied in terms of quality in consultations with nurses, but where the clinical results did not find differences between nursing and medicine.

The results obtained regarding prescriptions given by nurses indicate that patients maintained a perception of quality during the consultation or had a similar number of subsequent consultations for the same reasons as their medical colleagues, but with a lower number of prescribed medications. In the experimental group, an average of 1.79 medicines were prescribed, compared to the control group, where the average was 2.26. This is a clearly significant difference (*p* < 0.001). These results lead us to think that the NDM also has potential as an instrument to increase the efficiency and quality of health services in terms of pharmacological prescription. Despite this result, and as we have already commented previously, we believe it is necessary to point out that social changes and polymedication [56, 57] mean that health professionals are often confronted with the erroneous perception of some patients who measure quality of care from doctors or nurses based on the quantity of medicines prescribed, notwithstanding their best efforts to promote reasonable use of medication.

### Strengths and limitations

The main strength of this study is its design, since, to our knowledge, there are no recent experimental studies on nursing demand management in primary care emergency centres for minor health problems.

Regarding limitations, this study was carried out in a local context, so the results should be considered with caution. Furthermore, our study required considerable time to recruit the necessary sample. As this was voluntary work, the recruitment of participants and their follow-up, as well as the telephone surveys, had to be adjusted to the on-call shifts of the volunteer nurses and doctors.

## Conclusion

The results of the study suggest that the implementation of Nurse Demand Management (NDM) was a factor in improving the efficiency and quality of care in the Primary Care Emergency Centre (PCEC). The capacity of nurses for resolution has been shown by the results in the low rate of consultations for the same reason afterwards, a decrease in the prescription of medicines and incapacity for work, and a level of satisfaction comparable to the care provided by doctors.

These results should be considered with caution since the project was carried out in a small territory, specifically in the municipality of Mataró, in the autonomous community of Catalonia, Spain. As such, we consider that a second multi-centre experimental project is needed to analyse the potential of the NDM extrapolated to PC emergencies in a wider territory.

## Electronic supplementary material

Below is the link to the electronic supplementary material.

Supplementary Material 1

## Data Availability

The datasets used in this study are confdential but available upon request from the corresponding autor.

## Abbreviations

PC: Primary Care
PHAMEU: Primary Health Care Activity Monitor for Europe
ICS: Catalan Institute of Health
NIC: Nursing Interventions Classification
NOC: Nursing Outcomes Classification
NANDA: North American Nursing Diagnosis Association
ATIC: Architecture, Terminology, Interface-Nurse-Information and Knowledge
PCEC: Primary Care Emergency Centre
NDM: Nurse Demand Management
ARES-AP: Treatment Standards Harmonization Program for nurses in primary care
ECAP: Primary Care Clinical History software in Catalonia
TREND: Transparent reporting of evaluations with non-randomized designs
MAT: Andorran Screening Model
REDCap: Research Electronic Data Capture management system
IDIAPJGol: Jordi Gol University Institute for Research in Primary Health Care
EU: European Union
GDPR: General Data Protection Regulations
PIUC: Comprehensive Emergency Plan of Catalonia
