# Supplementary material for: Nurse management of minor problems in primary care emergencies: a non-randomized controlled trial
Source: BMC Nurs. 2025 Jan 24;24:87. doi: 10.1186/s12912-025-02729-2 (PMC11758717; doi:10.1186/s12912-025-02729-2)
Supplement: Supplementary file 1 — Supplementary Material 1 [file 12912_2025_2729_MOESM1_ESM.pdf]

Institut Català de la Salut

CUAP MARESME
Antonio Diaz Conde, Mataró, Barcelona, Spain.

## Satisfaction questionnaire EXPERIMENTAL GROUP

Research study "The implementation of nursing demand management as a factor for improving the quality of care in a primary care emergency center (CUAP)

*To be completed by the triage nurse and/or the GID consultation*

PATIENT CODING: E/_______

* The coding for the experimental group will always begin with the letter E + a non-repeated participant number between 1 and 156. To clarify any doubts you can contact the monitor of the study or the GID referent of the shift.

| Date of first visit:: | Professional GED | Reason for consultation: ○ Urinary problems ○ Upper airways ○ Skin lesions ○ Toothache ○ Gastrointestinal disorders |
|---|---|---|

Attendance rating 1 (not very satisfied) to 10 (very satisfied))

Any comments from the user?

*To be completed from 10-15 days after the first visit*

Attendance rating 1 (not very satisfied) to 10 (very satisfied)

Have you needed to carry out any reconsultation for the same reason? YES NO

Has the treatment prescribed in the GED consultation been effective to solve your health problem? YES NO

If you have had to consult again, the treatment prescribed in the GED consultation has been modified If you have had to consult again, has the treatment prescribed in the GED consultation been subsequently modified by a doctor to solve your health problem? YES NO

Do you think that your reason for consultation would have been better treated by a doctor? YES NO

How do you rate your attendance through the GID (1 -not very satisfied- to 10 -very satisfied-)

| | | | |
|---|---|---|---|
| At the level of agility in care at the first visit | ☐ | At the level of professionalism of the GED nurse | ☐ |
| At the level of effectiveness of the treatment prescribed in the GID consultation | ☐ | At the level of effectiveness of the treatment prescribed in the GID consultation | ☐ |

Institut Català de la Salut

CUAP MARESME
Antonio Diaz Conde, Mataró, Barcelona, Spain.

## Satisfaction questionnaire CONTROL GROUP

Research study "The implementation of nursing demand management as a factor for improving the quality of care in a primary care emergency center (CUAP)

*To be completed by the triage nurse and/or the GID consultation*

PATIENT CODING: E/_______

* The coding for the experimental group will always begin with the letter E + a non-repeated participant number between 1 and 156. To clarify any doubts you can contact the monitor of the study or the GED referent of the shift.

| Date of first visit: | Professional GeD | Reason for consultation: Urinary problems Upper airways Skin lesions Toothache Gastrointestinal disorders |
|---|---|---|

Attendance rating 1 (not very satisfied) to 10 (very satisfied)

Any comments from the user?

To be completed from 10-15 days after the first visit

Attendance rating 1 (not very satisfied) to 10 (very satisfied) ☐

Have you needed to carry out any reconsultation for the same reason? ◯ YES ◯ NO

Has the treatment prescribed in the MEDICAL VISIT consultation been effective to solve your health problem? YES NO

If you have had to consult again, the treatment prescribed in the GED consultation has been modified If you have had to consult again, has the treatment prescribed in the GED consultation been subsequently modified by a doctor to solve your health problem? YES NO

Do you think that your reason for consultation would have been better treated by a nurse? YES NO

How do you rate your attendance through the GID (1 -not very satisfied- to 10 -very satisfied-)

| | | | |
|---|---|---|---|
| At the level of agility in care at the first visit | ☐ | At the level of professionalism of the GED nurse | ☐ |
| At the level of effectiveness of the treatment prescribed in the GID consultation | ☐ | At the level of effectiveness of the treatment prescribed in the GID consultation | ☐ |
